# Mushroom Natural Products in Neurodegenerative Disease Drug Discovery

**DOI:** 10.3390/cells11233938

**Published:** 2022-12-06

**Authors:** Arjuna Abitbol, Brody Mallard, Evelin Tiralongo, Joe Tiralongo

**Affiliations:** 1 Institute for Glycomics, Gold Coast Campus, Griffith University, Southport, QLD 4222, Australia; 2 School of Pharmacy and Medical Science, Gold Coast Campus, Griffith University, Southport, QLD 4222, Australia

**Keywords:** neurodegenerative diseases (NDs), natural products (NPs), Alzheimer’s disease (AD), Parkinson’s disease (PD), multiple sclerosis (MS), Huntington’s disease (HD), nucleotide-binding oligomerisation domain- leucine-rich repeat- and pyrin domain-containing 3 (NLRP3), amyloid-β (Aβ), synuclein α (SNCA), polyglutamine (poly Q)

## Abstract

The variety of drugs available to treat neurodegenerative diseases is limited. Most of these drug’s efficacy is restricted by individual genetics and disease stages and usually do not prevent neurodegeneration acting long after irreversible damage has already occurred. Thus, drugs targeting the molecular mechanisms underlying subsequent neurodegeneration have the potential to negate symptom manifestation and subsequent neurodegeneration. Neuroinflammation is a common feature of neurodegenerative diseases such as Alzheimer’s disease, Parkinson’s disease, Huntington’s disease, and multiple sclerosis, and is associated with the activation of the NLRP3 inflammasome, which in turn leads to neurodegeneration. Inflammasome activation and oligomerisation is suggested to be a major driver of disease progression occurring in microglia. With several natural products and natural product derivatives currently in clinical trials, mushrooms have been highlighted as a rich and largely untapped source of biologically active compounds in both in vitro and in vivo neurodegenerative disease models, partially supported by successful clinical trial evaluations. Additionally, novel high-throughput methods for the screening of natural product compound libraries are being developed to help accelerate the neurodegenerative disease drug discovery process, targeting neuroinflammation. However, the breadth of research relating to mushroom natural product high-throughput screening is limited, providing an exciting opportunity for further detailed investigations.

## 1. Ageing & Neurodegenerative Diseases

Ageing is inevitable. Now more than ever, humans are living longer thanks to constant advances in modern medicine, our forever-growing understanding of the human body, and our knowledge of treating the diseases that affect us. However, several consequences can affect the quality of this extended time. During the process of ageing, the brain undergoes both structural and physiological changes that directly affect cognitive function and homeostatic processes [1]. Specifically, neurodegenerative diseases (NDs) seem to be an ever-more-frequent consequence of brain-related ageing [1]. 

Between the ages of 50 to 90, the average rate of atrophy in the human brain can equate to approximately 11% or 150 g, affecting both the structure and physiology of the brain itself [2]. Other changes include accelerated cortical thinning, hydrocephalus, leukoaraiosis, axonal loss, genomic instability, changes in proteostasis, dysfunction of the immune system, mitochondrial instability, and aberrations in the autophagic and lysosomal degradative pathways–many of which can be associated with the normal upkeep of the cellular environments within the central nervous system (CNS) [1,3,4,5,6,7,8]. 

The newly coined term “inflamm-ageing” describes a type of underlying and chronic age-related inflammation [1]. This inflamm-ageing process is thought to influence age-related pathologies dictating the type of ND one may acquire, the rate of disease progression, and even responsiveness to therapeutic interventions [1]. In the CNS, the drivers of inflamm-ageing are glial cells, whereby prolonged activation resulting in the production of inflammatory cytokines drives immune cell invasion and subsequent neurodegeneration [9]. In addition, phagocytic microglia play an apparent but not completely understood role in neuroinflammation and brain-immune dysregulation, where this could be a potential overlap in many of the most common NDs [10,11,12,13]. 

**Figure 1:**
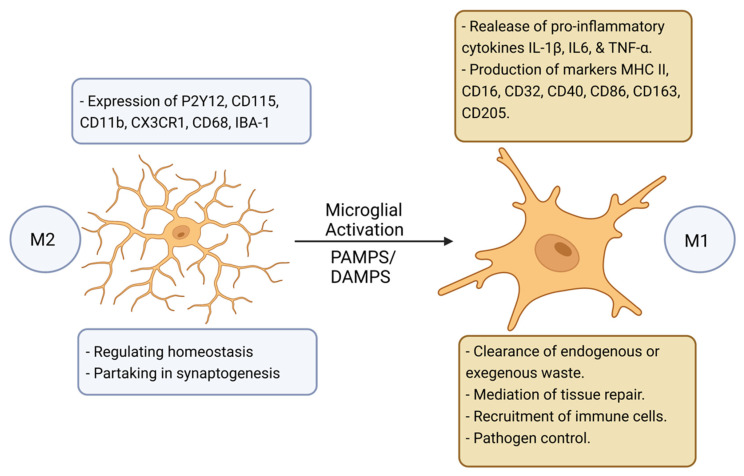
A schematic representation of the model proposed for microglial activation [15]. Microglia undergo a transition from M2 “resting” state to M1 “active” state which is induced by various molecular signals. Figure created with BioRender.com (accessed on 1 November 2022).

In terms of the CNS, microglia are the primary innate immune cells, serving to act as the first line of defense in response to pathogen-associated molecular patterns (PAMPs) and/or endogenous damage-associated molecular patterns (DAMPs) [14]. Microglia await activation from their quiescent “resting” state (M2) to their activated state (M1) by detecting minute changes in their immediate environment via surface receptors (Figure 1) [14,15]. Microglia are also involved in homeostatic-type tasks such as synaptogenesis and phagocytotic activities [12].

Activated microglia secrete proinflammatory cytokines and chemokines that help to recruit immune cells driving neuroinflammation [16,17,18]. In the context of NDs, physiological homeostasis between microglial activation and their return back to the M2 quiescent state is impeded; whereby, prolonged pro-inflammatory activation promotes the destruction of surrounding cells [15]. Moreover, through the process of ageing, microglia can endure phenotypic changes and become increasingly hypersensitive expressing more master histocompatibility complex II (MHCII) molecules which is linked to the increased expression of inflammatory cytokines such as IL-1β, further driving chronic underlying neuroinflammation [15].

### 1.1. Alzheimer’s Disease: Pathological Protein Accumulation Leads to Microglial Activation and Systemic Neuroinflammation

Alzheimer’s disease (AD) is prolific in the elderly and is characterised by the accumulation of mutant amyloid-β (Aβ) protein and neurofibrillary tangles (NFTs) in otherwise healthy neuronal cells of the entorhinal cortex and the hippocampus [19,20]. There is no cure or prevention for this disease, only drugs to treat the symptoms. However, research suggests that valuable drug targets may lie in the key components that drive immune dysfunction and neuroinflammation [21]. 

In the latter stages of AD, microglia also undergo phenotypic changes such as decreased phagocytic abilities and the absence of proinflammatory cytokine production, a type of cellular quiescence, promoting pathological protein accumulation [22]. Moreover, microglial activation is coupled with the presence of the characteristic Aβ plaque and NFT deposition, driving the explicit overexpression of the nucleotide-binding oligomerisation domain-, leucine-rich repeat- and pyrin domain-containing 3 (NLRP3) inflammasome complex within the microglia itself, also driving the cleavage of gasdermin forming cellular pores and initiating pyroptotic cell death [23,24]. 

More recently, monoamine oxidase-A (MAO-A) and MAO-B, which is involved in the catabolism of catecholamines in the frontal lobe, neo cortex, parietal cortex, occipital cortex, temporal cortex, cerebral cortex, and hippocampus, have been found to play a role in AD pathogenesis [25]. Studies suggest that immunoreactive microglia and astroglia have altered levels of MAO facilitating the abnormal cleavage, via APP, of Aβ peptide [26]. Additionally, altered levels in MAO-A and MAO-B showed increased neurotoxic monoamine metabolites promoting Aβ deposition where MAO inhibitors (MAOIs) have also show efficacy in treating AD-related neurodegeneration via this pathway [26]. Furthermore, MAO are found restricted to the mitochondrial membrane and is also related to the production of reactive oxygen species (ROS) reactive nitrogen species (RNS) further driving the activation of microglia and mitochondrial dysfunction [26].

### 1.2. Parkinson’s Disease and the Activation of the NLRP3 Inflammasome in Activated Microglia Are Drivers of Neuroinflammation

Several highly penetrative genetic variants, out of over 30 identified genes, play a significant role in determining the likelihood of therapeutic efficacy, disease onset, symptom manifestation, and pathogenesis in PD [27,28]. The most common gene variants in PD include mutations in synuclein alpha (SNCA), parkin RBR E3 ubiquitin-protein ligase (PRKN), protein deglycase-1(DJ-1), PTEN induced kinase 1 (PINK1), leucine-rich repeat kinase 2 (LRRK2), and vacuolar protein sorting ortholog 35 (VPS35), which all play a role in neuroinflammation, aberrations in synaptic transmission, neuronal cell degradation, and cognitive decline [27,28].

The SNCA gene variants are shown to be directly linked to neuroinflammation via the activation of the proinflammatory cytokine interferon-γ (IFN-γ) and regulatory resolvin D1, along with promoting changes in T-cell receptor expression and the up-regulation of master histocompatibility Class I (MHC I)-driving an autoreactive response with the T-cells [29]. Mutant α-syn is known to play a role in Lewy body formation in dementia, multiple system atrophy (MSA), the rare AD variant with a Lewy body proponent, and neurodegeneration with brain iron accumulation type I [30,31,32,33,34]. Furthermore, the affected brain regions of the substantia nigra and corpus striatum (where dopaminergic neurons reside) that are affected in PD, report extended activation of microglia which are suggested to drive the promotion of dopaminergic neuronal cell death due to the activation of the NLRP3 inflammasome complex, accompanied by the simultaneous increase in reactive ROS and nitrous oxide (NO) levels [15,35,36]. 

### 1.3. Activated Microglia Drives an Auto-Reactive Immune Response and Neuroinflammation in Multiple Sclerosis, but Also Cleans Debris Promoting New Tissue Growth

Multiple sclerosis (MS) is characterised by progressive demyelination and neuronal degradation along with systemic neuroinflammation in the CNS, present throughout the whole disease process, activating both the adaptive and innate immune system [37]. The etiology of MS is largely unknown; however, this disease has strong genetic and epigenetic components that are thought to play a role in disease onset and progression [37]. In MS, activated microglia are continually recruited into the CNS by the perpetual signaling of inflammatory cytokines and the presence of ROS and RNS [38]. Research suggests that the innate anti-oxidative mechanisms are unable to maintain the balance with the excessive amount of ROS and RNS generated, causing damage to lipids and proteins, being deleterious to cellular health, ending in cell death [39]. Furthermore, well known cell survival and gene expression regulators are also affected by the overproduction of ROS and RNS, for example, nuclear factor-erythroid 2-related factor 2 (Nrf2), nuclear factor-κB (NF-κB), and mitogen-activated protein kinases (MAPKs) [40,41,42]. 

In MS, microglia act as antigen-presenting cells recruiting T-cells by upregulating the expression of MHCII molecules [43]. When microglia are unable to be deactivated by Pellino E3 ubiquitin-protein ligase 1 (Peli1), Peli1 acts as a negative regulator of T-cell activation and thus further drives disease progression [44,45]. However, this is a double-edged sword as microglia cells also work both to remove the remnants of degraded myelin debris and the apoptotic cell remains, helping to promote the regeneration of lost tissues [46].

### 1.4. Huntington’s Disease

Huntington’s disease (HD) is characterised by the trinucleotide expansion of a CAG repeat found in exon 1 of the Huntingtin (HTT) gene [47,48,49,50]. This expansion encodes a stretch of polyglutamine (poly Q) in the N-terminal region of the protein, and physically manifests by the progressive atrophy of the basal ganglia, cortex, and hippocampus [47,48,49,50]. Progressive expansions of this gene through time, coincides with the perpetual decline in motor symptoms [51,52]. These termed *polyglutaminopathies* are unique to HD and other conditions such as the spinocerebellar ataxias (SCA) types 1, 2, 6, 7, and 17, as well as Machado-Joseph disease, dentatorubral pallidoluysian atrophy, and spinal and bulbar muscular atrophy X-linked type 1, which also manifest with some of the same physical symptoms as HD [53]. 

There is a lack of evidence definitively linking CAG expansions as the primary force driving pathogenesis in HD and further research suggests that the protein tau, as found in AD, can contribute to the associated chronic neuroinflammation driven by the characteristic microglial activation [54,55]. Like in other NDs, microglial activation is driven by the oligomerisation of the NLRP3 inflammasome complex, detectable via biomarkers such as cytokines IL-6 (a pro-inflammatory cytokine), IL-8 (recruitment of immune cells to the site of inflammation), and TNF-α (a pro-inflammatory cytokine) [56]. Furthermore, microglial activation seems similar between AD, PD, MS, HD and other NDs [12,13,14,57,58]. This overlap serves as an excellent potential target for drug discovery and development, whereby probing these molecular mechanisms will at a minimum provide us a greater understanding of the molecular pathways of these disease states.

## 2. Microglial Activation and NLRP3 Inflammasome Oligomerisation Drives Pyroptosis, Neuroinflammation, and Neurodegeneration

As previously mentioned, the drivers of inflamm-ageing and more specifically neuroinflammation in NDs are related to metabolic and phenotypic changes in neuroglia, specifically astrocytes, microglia and oligodendrocytes [59]. The resultant neurodegeneration is largely associated with pyroptosis (inflammatory programmed cell death), and inflammasome activation is directly linked to the hallmark pathology of abnormal protein aggregates [60,61,62]. 

**Figure 2:**
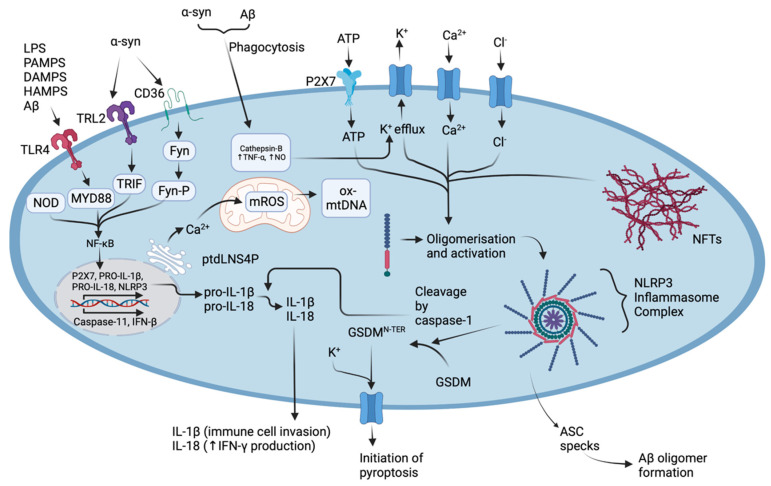
The molecular mechanisms underlying the activation of the NLRP3 inflammasome complex in AD and PD. Abnormal protein aggregates such as Aβ can selectively bind TLR4, SNCA can selectively bind TLR2 and scavenger receptor CD36, or both can be phagocytosed [67]. Phagocytosed Aβ and SNCA drive the production of cathepsins which increase K^+^ efflux, TNF-α and NO production, and NLRP3 oligomerisation [67,68]. Intracellular messengers such as MYD88, TRIF, and Fyn act to upregulate NF-κB which stimulates the production of proinflammatory cytokine pro-IL-1β, pro-IL-18, the production of NLRP3, and additionally increases the expression of caspase-11 and IFN-β [63,64,67,69,70,71]. The ER drives Ca^2+^ uptake in mitochondria, along with intracellular ion efflux and promotes mROS production, which in turn drives NLRP3 oligomerisation, also via the release of ptdLNS4P [4,20]. The activation of the inflammasome complex allows pro-caspase-1 to self-cleave forming active caspase-1 which can then induce the maturation of pro-IL-1β and pro-IL-18, cleave GSDMD initiating pyroptosis, drive the invasion of immune cells via increasing the expression of adhesion molecules, sensitise neutrophils to chemo-attractants, and stimulate vasodilation [67,69,70]. Figure created with BioRender.com (accessed on 1 November 2022).

The NLRP3 inflammasome is complex, assembled by the activation and localisation of several components: the “sensor” NACHT, LLR, and PYD domains-containing protein 3, the “adapter” apoptosis-associated speck-like protein containing a card (ASC), and “effector” caspase-1, all serving with various functions [63,64,65]. Inflammasome activation, ASC speck formation, and procaspase-1 activation require NIMA-related kinase 7 (NEK7), which is unique to the NLRP3 inflammasome; however, the details of the specific mechanisms, as depicted in Figure 2, are poorly understood [66]. 

As shown in Figure 2, mature caspase is responsible for the maturation of pro-IL-1β pro-IL18, and cleavage of gasdermin D (GSDMD)-all important factors contributing to the innate immune response and pyroptotic cell death; however, the details of the molecular pathways are still poorly understood [67]. Pyroptosis is carried out by swelling of the cell and cell lysis mediated by nerve injury-induced protein 1 (NINJ1), thus releasing the accumulated pro-inflammatory constituents extracellularly [69,72]. Pyroptosis stands as a characteristic feature between the common NDs and some studies suggest that the inducer of NLRP3-associated pyroptosis may be related to several processes, for example, (i) the binding of protein aggregates to Toll-like receptors (TLRs) on the cell surface, (ii) K^+^, Cl^−^, and Ca^2+^ efflux [68,73,74], (iii) disassembly of the trans-Golgi network, (iv) migration of the inactive complex via HDAC6-dynein machinery to the microtubule organising center (MTOC), and (v) localisation of NEK7 [75,76]. Furthermore, the molecular mediators of the NLRP3 inflammasome, and that of the pyroptosis, could serve as novel targets in the development of new drugs against NDs. By perturbating these targets, it is likely that drugs will treat the underlying causes of disease progression and neuroinflammation, rather than specifically treating the disease symptoms when substantial neurodegeneration has occurred.

## 3. Natural Products: Drug Discovery and Clinical Trials in Neurodegenerative Disease’s

NPs serve as a rich source of biologically active compounds, with many entering clinical trials studies for a multitude of diseases, as well as serving as molecular scaffolds to many drugs. Importantly, in the context of this review, many have entered clinical trials for NDs such as AD (Table 1). One of the most successful drug candidates, sodium oligomannate (GV-917) an acidic linear oligosaccharide, has finished Phase II clinical trials in China proving to be safe and well tolerated [77]. GV-917 is now in phase III, evaluating longer duration with tighter criteria regarding participant inclusion and exclusion [77]. Additionally, GV-917 has been assessed for its role in the remodeling of gut microbiota, the modulation of neuroinflammation via peripheral immune activation, and the regulation of the abnormal amino acid production found in AD pathology [78]. In this study, Wang et al., (2020) found that microglial activation in the brain is modulated by infiltration of T-helper (Th1) cells in the context of gut dysbiosis and that GV-917 “reconditioned” gut microbiota in turn reducing Th1-associated neuroinflammation in a mouse model-opening the potential for anti-AD drug development in the carbohydrate-NP space [78].

The drug ALZT-OP1 (Table 1) contains the non-selective cyclooxygenase (COX) inhibitor ibuprofen and the well-known mast cell stabilizer cromolyn sodium, a chromone and synthetic structural derivative of the NP fisetin [80]. In a preclinical study, ALZT-OP1 was shown to interfere with the oligomerisation of abnormal Aβ protein in an in vitro microglia model in addition to an in vivo transgenic mouse model [80]. The authors suggested that the mechanism of action is via moving microglia out of a pro-inflammatory and neurotoxic state, into an increased phagocytic and neuroprotective state [80]. Ongoing phase III trials are being conducted in both America and Australia [94].

J-147 is another promising drug acting as a neuroprotective compound that has been suggested to reverse cognitive impairment, slow the processes underlying ageing, and facilitate the aversion to age-associated maladies [82,95,96,97,98]. J-147 is a synthetic derivative of the NP curcumin and its main target is thought to be via the upregulation of AMPK, which in turn induces the phosphorylation of acetyl-CoA carboxylase 1 (ACC1), directly influencing mitochondrial stability [83]. Furthermore, J-147 inhibits the degradation of acetyl-CoA, leading to acetyl-CoA build-up, which has a neuroprotective effect [83]. This pathway and its targets could be further probed as a way to modulate mitochondrial instability in future ND drug development.

Epigallocatechin-gallate (EGCG) (Table 1), a polyphenol extracted from green tea, has been found to have a wide range of biological activities in in vivo AD mouse models [99]. EGCG decreases the amount of Aβ accumulation by actions through α-secretase, which cleaves γ-secretase, the enzyme mostly responsible for the formation of abnormal Aβ peptides [99]. EGCG also inhibits GSK3β expression, a key kinase in tau-hyperphosphorylation and the formation of NFTs, in the MC65 human neuronal cell line [100]. EGCG induces other overarching effects such as ameliorating oxidative stress, anti-apoptosis, inhibiting acetylcholine-esterase activity, decreasing synaptic dysfunction, and increasing pro-NGF and NGF levels, leading to a decrease in neuroinflammation [78,101,102,103,104,105,106]. Furthermore, in a 2016 Downs syndrome-associated AD clinical trial, the EGCG supplement exhibited increased cognitive improvements in comparison to the placebo [107]. There are no further trials currently registered with EGCG.

ALZ-801 (Table 1) is probably the most efficacious drug undergoing AD clinical trials at present. Isolated from seaweed, ALZ-801 is the pro-drug to tramiprosate, an NP and derivative of the amino acid taurine, that presents with improved tolerability and decreased pharmacokinetic variability [108,109]. ALZ-801 is among the first disease-modifying drugs used to treat APOE4 (homozygote or heterozygote) gene variant carriers [110], and is currently in phase III clinical trials. ALZ-801 is believed to block Aβ oligomer formation, without binding to plaques, which can be detected by analysing inflammatory CSF biomarkers [111]. 

An underutilized source of biologically significant NPs, mushrooms are known to have the potential to serve as a great source of compounds that could not only be used to elucidate ND-associated pathways but also potentially serve as the molecular scaffolds in drug development. In the final part of this review, we will focus on the significance of mushroom NPs in ND drug discovery with emphasis on the potential of Australian native mushrooms to be explored as a superior untapped source of medically relevant NPs.

## 4. Mushroom Natural Products: Their Relevance in Ameliorating Neuroinflammation and Contribution to Drug Development for Neurodegenerative Diseases

Mushroom NPs are gaining momentum in ND research, with countless studies over the last decade highlighting the relevance of mushroom NPs and their ability to modulate a multitude of various disease states. Recently, mushroom NPs have been investigated as modulators of neuroinflammation as well as serving as excellent tools for elucidating the molecular processes contributing to disease pathogenesis (Table 2). 

**Figure 3:**
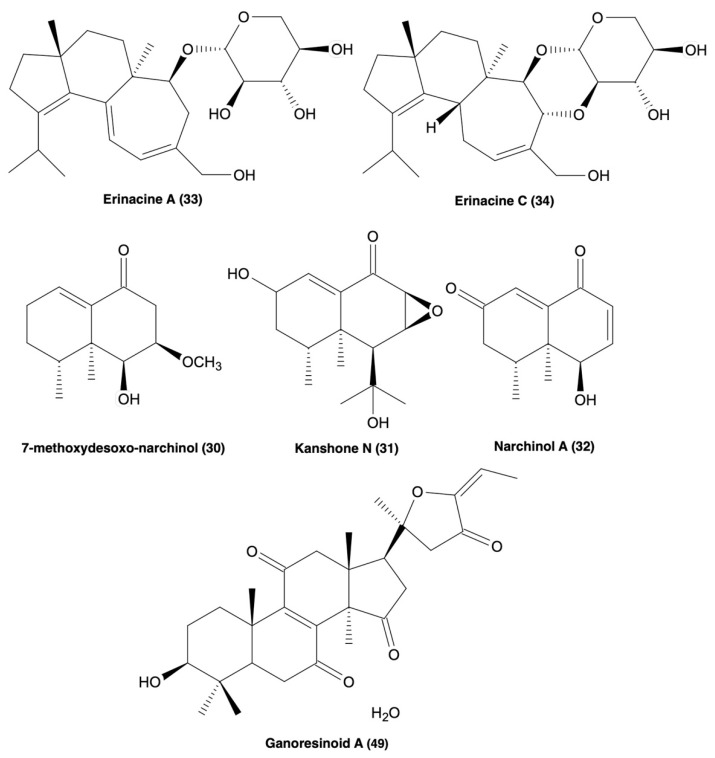
Molecular structures of erinacine A and C (**33** & **34**), 7-methoxydesoxo-narchinol (**30**), kanshone N (**31**), narchinal A (**32**), and ganoresinoid A (**49**), all of which possess anti-neuroinflammatory processes [119,120,121,124].

Many mushroom species have had their primary and secondary metabolite profiles extensively investigated, for example, *C. millitaris, I. obliquus, C. africanus, H. erinaceous*, and various Ganoderma species (Table 2). These species provide examples that mushroom NPs can alleviate neuroinflammation-mediated cell death, increase abnormal protein clearance, and promote anti-neuroinflammatory effects in both animal and cell models, without displaying cytotoxicity [112,113,114,116,117,118,119,120,121,122,123,124,125,126,127,128]. As seen in Table 2, a wide variety of compounds have been at the forefront of recent ND research, including but are not limited to polysaccharides, variations of di, tri, sesqui, and lanostane-based terpenoids, and phenols. At the forefront of mushroom NP investigations in neurodegeneration is *H. erinaceous*. Erinacine A (Figure 3), a compound isolated from the mycelia of *H. erinaceous*, has been extensively investigated for its neurotropic and neuroprotective effects and is being evaluated in clinical trials for its mitigation of AD symptoms [93], and we will further discuss this compound later in this review. Mushroom NPs also show great potential for both probing and elucidating the poorly understood neuroinflammatory pathways, as well as serving as potential scaffolds for drug development.

### 4.1. Mushroom Derived Polysaccharide Natural Products and Extracts

Polysaccharide extracts from *V. velutipes* have been reported to exhibit significant effects in vitro and in vivo, restoring acetylcholine esterase (AChE) activity and reversing spatial memory deficits in scopolamine impaired rats, as well as increasing the levels of expression of connexin-36 and p-CaMKII, involved in the synthesis and secretion of neurotransmitters (Table 2) [112,113]. Similarly, a polysaccharide extract from the mushroom *G. frondosa* restored escape latency time and improved cognition in APP/PS1 mice at concentrations between 5–10 mg/kg (Table 2) [114]. In addition, *G. frondosa* polysaccharides ameliorated histological and necrotic morphology in the isolated mice hippocampal cells, decreased Aβ pathology (per mm^2^), increased microglial and astrocyte activation, and increased microglial mediated clearance of pathological Aβ [114]. These findings all indicate that polysaccharide extracts should be further investigated for their ability to modulate neuroinflammation-associated pathways.

### 4.2. Mushroom Derived Terpenes

Another class of NPs, the cyathane diterpenoids, have been highlighted for their biological activities in both in vivo and in vitro ND models. New NPs isolated from mushroom *C. africanus,* the polyoxygenated neocyathins K–R (**4**–**11**) and three known congeners (**12**–**14**), have been shown to increase the production of neurite bearing cells at a concentration between 1–25 μM in the presence of NGF (20 ng/mL) in PC-12 cells, along with no cytotoxicity in both PC-12 cells and BV2 microglia (Table 2) [116]. Additionally, compound 50 inhibited the inducible nitric-oxide synthase (iNOS) enzyme at IC_50_ = 19.8 μM supported by molecular docking studies [116]. Another study described four additional bioactive cyathane diterpenoids, cyanthane I (**15**), (12*R*)-11*α*,14*α*-epoxy-13*α*,14*β*,15-trihydroxycyath-3-ene (**16**), cyathin O (**17**), and allocyafrin B_4_ (**18**) (Table 2) [117]. Compounds **15**–**18** abolished iNOS expression in Aβ_1-42_-induced and LPS-induced BV2 microglia, supported by molecular docking studies [117]. Compounds **15** and **18** strongly inhibited COX-2 expression in the Aβ_1-42_ and LPS-induced BV2 microglia, also supported by molecular docking, and also displayed no cytotoxicity in BV2 microglia [117]. Additionally, eleven more biologically active cyathane diterpenoids have been isolated from *C. africanus*, cyafricanins A–K (**19**–**29**) (Table 2) [118]. All the cyafricanins displayed no cytotoxicity between 5–100 μM and increased neurite-bearing cells in the PC-12 cell line in the presence of NGF (20 μg/mL) [118]. Compound **29** suppressed COX-2 expression, **20** suppressed iNOS expression, and **19** and **20** suppressed NO production, all in LPS-induced BV2 microglia [118]. The species *H. erinaceus* has also been recently found to contain two biologically active cyathane diterpenoids, erinacine A (**33**) and erinacine C (**34**) (Table 2 and Figure 3) [120,121]. **33** inhibited iNOS and NO production and decreased TNF-α expression at 20 μM in LPS-induced astrocytes [120,121]. Compound **33** also decreased the expression of tyrosine hydroxylase, JNA, and NF-κB expression in N2a cells, while exhibiting no signs of cytotoxicity [120,121]. In vivo, **33** also improved motor function and decreased the expression of inflammatory markers. Compound **34** showed decreased cell viability <50% at 10 μM, but not between 0.1–2.5 μM, along with a decrease in the expression of iNOS, IL-6 and TNF-α, IκB- α phosphorylation, and increased Nrf2 expression in LPS-induced BV2 microglia [120,121]. Cyathane diterpenoids show great potential with both in vitro and in vivo anti-neuroinflammatory effects, suggesting more research should be done to investigate structure-activity relationships and derivatisation to determine the moieties responsible for the bioactivity of these compound types.

Other isoprene unit containing NPs such as di, tri, and sesquiterpenes are indicated to also have significant pharmacological activities. A 2018 study reported bioactive sesquiterpenoids isolated from the mushroom *N. jatamansi*, 7-methoxydesoxo-narchinol (**130**), konshone N (**131**), and nardosdaucanol (**132**) (Table 2 and Figure 3) [119]. Compounds **130**, **131**, & **135** exhibited anti-inflammatory effects in LPS-induced BV2 microglia by decreasing the expression of iNOS, PGE2, COX-2, IL-12, IL-1β and TNF-α, and also by blocking the translocation of p65/p50 and phosphorylation of Iκ-B-α, with no cytotoxicity [119]. Therefore, isoprene unit-containing compounds may also be of significance in ND drug design and discovery.

### 4.3. Mushroom Derived Lanostanoids

Lanostanoid-type compounds constitute another family of biologically active compounds targeting neuroinflammation (Table 2). A recent study highlighted new biologically active polyoxygenated lanostanoids, inonotusols H–N (**39**–**41**), isolated from the mushroom *I. obliquus* [122]. Compounds **35**, **36**, **39**, & **40** showed the strongest NO inhibition (IC_50_ = 2.32–9.17 μM) in LPS-induced BV2 microglia, further supported by molecular docking studies that highlighted **36** and **39** as strongly interacting with iNOS. The same compounds displayed strong inhibition to iNOS expression but had little effect on COX-2 [122]. Lanostanoid-type compounds have also been isolated from two *Ganoderma* species, *G. obriforme* and *G. resinaceum*, along with several other types of known compounds mostly consisting of triterpenoids and meroterpenoids (Table 2) [123,124]. *G. obriforme*’s 3,4-*seco*-27-norlanostenoids, and ganorbifates C–I (**42**–**48**), all inhibited the production of NO and showed no cytotoxicity in LPS-induced BV2 microglia [123]. *G. resinaceum* also contains bioactive lanostane triterpenoids ganoresinoids A and B (**49** & **50**) [124]. Compounds **49** (Figure 3) and **50** (Table 2) strongly inhibited NO production in LPS-induced BV2 microglia, also displaying no cytotoxicity [124]. However, **49** was noted to have extremely strong inhibitory effects on the expression of TNF-α, IL-1β, IL-6, iNOS, COX2, TLR4, and NF-κB, in addition to ameliorating ROS-induced MMP dysfunction and apoptosis [124]. Compound **49** also increased phosphorylation of Akt and GSK-3β and increased the expression of HO-1, NQO-1, and Nrf2 in SH-SY5Y cells [124]. In addition to substantial evidence supporting a multitude of biologically active constituents in Ganoderma species, lanostanoid-type compounds may represent exceptional scaffolds in ND drug design and development.

### 4.4. Other Mushroom Derived Bioactive Natural Products

Other examples of mushroom NPs, with relevance to neuroinflammation in NDs, include the 3′-nucleoside analogue cordycepin, isolated from the extensively investigated mushroom species *C. millitaris,* a polysaccharide extract from *F.* velutipes, and phenolic-based extracts from the species *M. elata*, *S. luteus, P. eryngii, C. gunnii,* and *F. velutipes* (Table 2) [112,113,126,127]. These compounds and extracts exhibit a wide variety of biological activities ranging from antioxidant activity, ferrous ion chelating activity, anti-apoptosis, ROS reversing activity, restoration of AChE activity, inhibition of the formation of p-tau in vivo, and the regulation of Ca^2+^ efflux associated neurotoxicity and downstream neuroinflammation [112,113,126,127]. 

Ideally, the high-throughput screening of mushroom NPs and extracts would be the most efficient method for identifying bioactive constituents with relevance to NDs. The current limitation in the field is the lack of high-throughput methods targeting neuroinflammation. As previously noted, research suggests an overlap between NDs and neuroinflammation that could potentially serve as a multi-targeted scheme (MTS) in drug development [129,130]. New assays are being developed to target these neuroinflammatory pathways (Figure 2) in vitro, with mitochondrial dysfunction, the production of inflammatory cytokines, ROS, and NOS, and uncontrolled Ca^2+^ efflux leading to neurodegeneration, being of great importance in the development of new screening assays [131,132]. In the next section we will discuss how these pathways are being targeted using conventional tests as well as newly developed high-throughput assays.

## 5. High-Throughput Assays Which Can Be Used to Screen Mushroom NPs and Extracts

As previously mentioned, mushrooms are a good source of new NPs for the treatment of NDs, however, to develop the field further, improved high-throughput screening methods are required to assess large compound libraries. With common assays outlined in Table 3, the standard cytotoxicity screening method, the 3-(4,5-dimethylthiazol-2-yl)-2,5-diphenyltetrazolium bromide (MTT) assay, serves as a gold standard for detecting mitochondrial dysfunction-associated cell viability [133]. As seen in Figure 2., mitochondria will produce mROS promoting NLRP3 inflammasome oligomerisation [20]. This mROS production is directly related to mitochondrial dehydrogenase activity which serves as the basis for the MTT assay [133]. The MTT assay is inexpensive and can be used as a first step in the screening of mushroom NPs or extracts in a high-throughput manner [134]. Additionally, mitochondria are the primary suppliers of ATP to neurons and their dysfunction in the early stages of neurodegeneration can be investigated through the analysis of ion efflux and ROS production, as shown in the NLRP3 inflammasome activation pathway in Figure 2, making them an invaluable target to probe neuroinflammatory pathways [20,82].

The inhibition of NO production or iNOS expression in vitro can be indicative of the reduction in neuroinflammatory cytokines such as TNF-α and IL-1β [139]. High levels of NO in glia are directly linked to neuronal cell death via mitochondrial cytochrome oxidase, a transmembrane proton pump associated with ATP production [140]. NO production has also been found to induce the depolarisation of neurons, the release of glutamate resulting in Ca^2+^ efflux, another driver of neuroinflammation, and the inhibition of oxidative phosphorylation resulting in oxidative stress [141,142]. 

As seen in Figure 2, IL-1β is expressed driving the transcription of iNOS genes [143], and as activated microglia express NO during neuroinflammation, in vitro NO expression can be detected with simple reagents such as sulfanilamide and N-1-naphthylethylenediamine dihydrochloride (NED) (also known as Greiss reagent), which can be easily applied to a multi-well high-throughput screening method [15]. Detecting NO production is a cheap alternative that could be used instead of the more expensive ELISA-based cytokine assays which could be used to probe IL-1β secretion.

Recently, Jiarong et al., (2022) reported on the development of a highly selective fluorescent probe, hemicyanine-coumarin hybridization fluorophore probe (HCCP), that targets monoamine oxidase-A (MAO-A) for the real-time imaging and analysis of living cells and tissues [136]. As mentioned in Section 1.1, MAO-A is a mitochondrial membrane-bound modulatory enzyme that acts upon degradation and maintains the balance of aminergic neurotransmitters [136]. MAO-A is linked to a variety of neurodegenerative and neuropsychiatric diseases and disorders, also serving as the basis for many drug mechanisms [136,144,145,146]. In AD, MAO-A is upregulated and drives abnormal Aβ cleavage by APP and also by the abnormal protein aggregate α-syn found in PD. Jiarong et al. (2022) suggests that the mitochondria is an invaluable target to probe for indications of neuroinflammation and that the HCCP assay can be used as a high-throughput method for screening NPs and extracts [136]. Jiarong et al. (2022) screened 210 “traditional Chinese medicines” in a high-throughput manner and identified evocarpine, from the plant *Evodia rutaecarpa*, as a potent inhibitor of MAO-A [136]. Evocarpine was then found to also inhibit iNOS expression, inhibiting the production of NO in LPS-induced BV2 microglia [136]. Furthermore, as neuroinflammation points to mitochondrial dysfunction, this study confirmed that HCCP can be used to explore the role of MAO-A expression in mitochondria in a high-throughput manner.

As previously mentioned, NLRP3 inflammasome activation is gaining increasing interest as a drug target and in screening assays as it is a main driver of pyroptosis as described in Section 2 for microglial activation and as depicted in Figure 2 with the cleavage of GSDM and pore formation [65,70,147]. Sohaib et al. (2021) have developed a new high-throughput screening assay that can be used to test inhibitors of NLRP3 inflammasome activation using immortalized bone marrow-derived macrophages (iBMDMs) [137]. Using the fluorescent mCherry tag, they were able to detect and visualise ASC speck formation, which occurs after NLRP3 activation [137]. Additionally, they screened 81,000 compounds finding several “hits” that inhibited various molecular paths and mediators such as heat shock protein 90 (HSP90), Janus kinase (JAK), and IKB kinase (IKK-β). These hit molecules were then tested in more specific NLRP3-associated processes such as in the detection of active caspase-1, pyroptotic cell death, and IL-1β production. Furthermore, the findings Sohaib et al. (2021) presents as a new method for identifying inhibitory compounds of the NLRP3 inflammasome.

In addition, Figure 2 shows that abnormal protein aggregates are at the initiation stage of neuroinflammation, binding to TLRs and scavenger receptors, and activate a cascade of intracellular molecular mediators that drive the assembly of the NLRP3 inflammasome, the production of pro-inflammatory cytokines, and induce the pyroptotic state. McClure et al. (2019) developed a high-throughput amyloid thioflavin competitive binding optical assay (HATCO) that detects the fluorescent dye thioflavin-T, which when unbound to the Aβ peptide emits weak fluorescence with a λ_max_ at approximately 440–445 nm, but when bound to the Aβ peptide, its fluorescence increases to a λ_max_ of 485 nm [138]. This assay can be performed in a 384-well plates and thus represents a potentially invaluable tool for the screening of compound or extract libraries [138]. 

## 6. Conclusions

Research in the field of NDs is multi-faceted and complex, with developments ranging from sourcing, identifying, and isolating new medically significant compounds from organic specimens, to developing new assays that simulate the complexities of these ND states to facilitate the identification of medically significant compounds. As outlined, neuroinflammation can be utilised as an MTS contributing to neurodegeneration across the most common NDs. Neuroinflammatory pathways such as NLRP3 inflammasome activation serve as important targets for drug discovery and development. Additionally, as neuroinflammation is consistent throughout neurodegeneration process, with research showing that it significantly contributes to ND pathogenesis, drug development in this area could be the key to fundamentally halting disease progression and eliminating symptom manifestation.

In terms of drug design and development, many NPs and NP derivatives are being assessed in clinical trials against the most common NDs. NPs have long served as a rich source of biologically active compounds relevant to many disease treatments. More specifically, mushrooms seem to have enormous, yet unused potential as a source of biologically active compounds, especially in the realm of drug discovery and development for NDs. NPs such as erinacine A provide examples of a mushroom NP currently undergoing clinical trials, with many other compounds such as polysaccharides, terpenes, and lanostanoids, also being found to display neuroprotective effects in vitro and in vivo. Future research should be directed towards developing more high-throughput screening methods for mushroom NPs focusing on neuroinflammation.

## Figures and Tables

**Table 1:** Examples of natural products or natural product derivatives that are currently in clinical trials for Alzheimer’s disease.

Drug	Class, Composition and Origin	Mechanism of Action	Clinical Trial Status	Ref.
GV-917 (sodium oligomannate)	Acidic linear oligosaccharides that are found in marine brown algae	Suggested to recondition gut microbiota and alter peripheral immune system response underlying AD pathogenesis and penetrates BBB through GLUT1 destabilising Aβ fibril formation via forming non-toxic monomers	Active (phase 3)	[78,79]
ALZT-OP1	Family of chromones. The drug is a mixture of Cromolyn (a synthetic derivative of the natural product khellin) and ibuprofen	Mast cell stabilizer suggested by decreasing Ca^2+^ efflux driven granulation and microglial activation modulator	Completed (phase 1)	[80,81]
J-147	Phenyl hydrazide derivative of natural product curcumin	Activation of AMPK and stabilisation of AMPK/ACC1 signaling in mitochondria	Completed (phase 1)	[82,83,84]
Epigallocatechin-gallate (EGCG)	Polyphenol/catechin is commonly found in green tea	Induces α-secretase expression and decreased neuroinflammation by decreasing the expression of TLR4 in animal models	Completed (phase 4)	[85,86,87,88,89]
ALZ-801 (Valiltramiprosate)	Homotaurine is a modified amino acid commonly found in seaweed	The prodrug acts as an Aβ aggregation inhibitor with efficacy in APOE4 variants (heterozygotes < homozygotes)	Active (phase 2/3)	[90,91,92]
Erinacine A	Cyathin diterpenoid isolated from the mycelia of the mushroom *H. erinaceus*	Upregulate NGF gene expression, neurotrophic and neuroprotective activities	Completed (N/A)	[93]

**Table 2:** Examples of mushroom natural products and extracts reducing neuroinflammation in in vitro and in vivo neurodegenerative disease models.

Compound/Extract	Bioactivity	Cells/Model/Assay	Ref.
**Polysaccharides**			
Polysaccharide extracts	↑Spatial memory and cognition	MWM test in rats	[112,113]
	Restoring AChE levels	AChE activity assay kit	
	↑Connexin 36 & p-CaMKII expression	Anti-antibody detection kit	
Derived polysaccharide extract from maitake (PGM)	PGM (5 mg–10 mg/kg) ↑escape latency time and cognition	APP-PS1 mice	[114]
	PGM ameliorated histological and necrotic morphology, ↓Aβ/mm^2^ pathology, ↑microglial and astrocyte activation, and microglial mediated Aβ clearance	APP/PS1 mice isolated hippocampal cells	
**Terpenes**			
Ganomycin C (**1**), ganoresinain A (**2**), ganotheaecoloid G (**3**)	**1**, **2**, and **3** ↓glutamate-induced neurotoxicity	SH-SY5Y cells	[115]
New neocyathins K–R (**4–11**), & 3 known congeners: cyathin V, (12 S)-11α,14α-epoxy-13α,14β,15-trihydroxycyath-3-ene, & allocyathin B_2_ (**12–14**)	**4–14** no cytotoxicity (10 μM)	BV2 microglia & PC-12 cells.	[116]
	**4–14** ↑In neurite-bearing cells (1–25 μM) with NGF (20 ng/mL) in PC-12 cells	PC-12 cells	
	**14** ↓iNOS (IC_50_ = 19.8 μM)	BV2 microglia & molecular docking	
Cyanthane I (**15**), (12R)-11α,14α-epoxy-13α,14β,15-trihydroxycyath-3-ene (**16**), cyathin O (**17**), allocyafrin B_4_ (**18**)	**15–18** ↓NO suppression via iNOS & no cytotoxicity	Aβ_1-42_-induced & LPS-induced BV2 microglia, molecular docking, and Western blotting	[117]
	**15**, **16**, & **18** abolished iNOS expression	Aβ_1–42_-induced BV2 microglia and molecular docking	
	**15** & **18** ↓COX-2 expression in BV2 cells supported by molecular docking		
Cyafricanins A–K (**19–29**)	**19–29** (5–100 μM) + NGF (20 μg/mL) increased neurite-bearing cells & had no cytotoxicity	PC-12 cells	[118]
	**29** ↓COX-2 expression, **20** ↓iNOS expression, & **19** & **20** ↓NO production	LPS-induced BV2 cells	
7-methoxydesoxo-narchinol (**30**), Kanshone N (**31**), narchinol A (**32**)	**30–32** ↓iNOS, PGE_2_, COX-2, IL-12, IL-1β, TNF-α expression & ↑IL-10, blocked p65/p50 translocation and phosphorylation of Iκ-B-α & displayed no cytotoxicity	LPS-stimulated BV2 microglia	[119]
Erinacine A (**33**), erinacine C (**34**)	**33** ↓iNOS & NO (20 μM)	LPS-induced BV2 microglia	[93,120,121]
		LPS-stimulated astrocytes	
	**33** ↓TNF-α expression	N2a cells	
	**33** showed no cytotoxicity (1 mg/mL), ↓tyrosine hydroxylase, JNA, and NF-κB expression		
	**33** ↓inflammatory cytokine expression and ↑ motor and cognitive ability	LPS-induced mouse model	
	**34** ↓cell viability <50% (10 μM), but not at 0.1–2.5 μM. ↓iNOS, NO, IL-6, & TNF-α, P-IκB-α, and ↑Nrf2 expression	LPS-induced BV2 microglia	
**Lanostanoids**			
Inonotusols H–N (**35–41**)	**39–40** no cytotoxicity (25 μM)	BV2 microglia	[122]
	**35**, **36**, **39**, & **40** ↓NO (IC_50_ = 2.32–9.17 μM)	LPS-induced BV2 microglia	
	**36** & **39** ↓iNOS (50 μM)	LPS-induced BV2 microglia, Western blotting, and molecular docking	
Ganorbifates C–I (**42–48**)	**89–95** ↓NO, **89** had the strongest (IC_50_ = 4.37 μM)	LPS-induced BV2 microglia	[123]
New: Ganoresinoids A & B (**49 & 50**)	**96** & **97** ↓NO	LPS-induced BV2 cells	[124]
	**96** has no cytotoxicity at 10 μM, ↓TNF-α, IL-1β, IL-6, iNOS, COX2, TLR4, and NF-κB expression, and ameliorated ROS-induced MMP dysfunction and apoptosis	LPS-induced BV2 microglia	
	**96** ↑P-Akt and P-GSK-3β and [125] ↑HO-1, NQO-1, and Nrf2 expression	SH-SY5Y cells	
**Misc/extracts**			
Cordycepin	Cordyecepin ↓apoptosis, ROS-induced neuronal death, Ca2^+^ efflux, I_Ca_ dysfunction and resultant neurotoxicity via A_1_-R, AChE activity, and p-tau formation	Aβ_25–35_-induced rat hippocampal neurons	[126]
Phellxinye A (**51**), Inonotphenol A (**52**)	**51** & **52** have antioxidative capacity	DPPH and FRAP assay	[125]
	**52** ↓apoptosis and MMP dysfunction	H_2_O_2_-induced apoptosis model using SH-SY5Y cells and fluorescent markers Hoechst 33258 and JC-1, respectively	
MeOH extracts (**53–57**)	Weak ferrous ion chelating activity	FCA assay	[127]
	Antioxidant capacity	Trolox equivalent assay	
	Ferric reducing antioxidant power	Ferric ion reducing antioxidant power assay	

**Table 3:** Examples of assays that can be used to screen for natural products or extracts for anti-neuroinflammatory activity.

Assay	Sample Type	Target	Limitations	Size/High-Throughput	Ref.
MTT assay	Cells	Mitochondrial dehydrogenase dysfunction	Cells only, cannot be performed on tissue.	96-well plate.	[133]
ELISA-based cytokine expression assay	Cells, tissues	Detection of cytokine expression	Coefficient of variation of 15%	96-well plate.	[135]
Greiss Test	Cells, tissues	NO production	Limited by NO concentration > 5 μM	96-well plate.	[15]
HCCP assay	Cells, tissues	Mitochondrial MAO-A	Background fluorescence interference	96-well plate.	[136]
ASC speck detection assay	Cells	NLRP3-dependant ASC speck formation	Cytotoxicity in long treatments	384-well plate.	[137]
HATCO assay	Brain lysate containing Aβ peptide	Aβ-binding molecules	DMSO solvent up to 5% of the assay’s volume	384-well plate.	[138]

## Data Availability

Not applicable.
